# Origins of scale invariance in vocalization sequences and speech

**DOI:** 10.1371/journal.pcbi.1005996

**Published:** 2018-04-16

**Authors:** Fatemeh Khatami, Markus Wöhr, Heather L. Read, Monty A. Escabí

**Affiliations:** 1 Biomedical Engineering, University of Connecticut, Storrs, Connecticut, United States of America; 2 Behavioral Neuroscience, Experimental and Biological Psychology, Faculty of Psychology, Philipps-University of Marburg, Marburg, Germany; 3 Center for Mind, Brain, and Behavior (CMBB), Philipps-University of Marburg, Marburg, Germany; 4 Department of Psychological Sciences, University of Connecticut, Storrs, Connecticut, United States of America; 5 Electrical and Computer Engineering, University of Connecticut, Storrs, Connecticut, United States of America; University of California at Berkeley, UNITED STATES

## Abstract

To communicate effectively animals need to detect temporal vocalization cues that vary over several orders of magnitude in their amplitude and frequency content. This large range of temporal cues is evident in the power-law scale-invariant relationship between the power of temporal fluctuations in sounds and the sound modulation frequency (*f*). Though various forms of scale invariance have been described for natural sounds, the origins and implications of scale invariant phenomenon remain unknown. Using animal vocalization sequences, including continuous human speech, and a stochastic model of temporal amplitude fluctuations we demonstrate that temporal acoustic edges are the primary acoustic cue accounting for the scale invariant phenomenon. The modulation spectrum of vocalization sequences and the model both exhibit a dual regime lowpass structure with a flat region at low modulation frequencies and scale invariant 1/*f*^2^ trend for high modulation frequencies. Moreover, we find a time-frequency tradeoff between the average vocalization duration of each vocalization sequence and the cutoff frequency beyond which scale invariant behavior is observed. These results indicate that temporal edges are universal features responsible for scale invariance in vocalized sounds. This is significant since temporal acoustic edges are salient perceptually and the auditory system could exploit such statistical regularities to minimize redundancies and generate compact neural representations of vocalized sounds.

## Author summary

The efficient coding hypothesis posits that the brain encodes sensory signals efficiently in order to reduce metabolic cost and preserve behaviorally relevant environment information. In audition, recognition and coding depends on the brain’s ability to accurately and efficiently encode statistical regularities that are prevalent in natural sounds. Similarly, efficient audio coding and compression schemes attempt to preserve salient sound qualities while minimizing data bandwidth. A widely observed statistical regularity in nearly all natural sounds is the presence of scale invariance where the power of amplitude fluctuations is inversely related to the sound amplitude modulation frequency. In this study, we explore the physical sound cues responsible for the scale invariant phenomenon previously observed. We demonstrate that for animal vocalizations, including human speech, the scale invariant behavior is fully accounted by the presence of temporal acoustic edges that are largely created by opening and closing of the oral cavity and which mark the beginning and end of isolated vocalizations. The findings thus identify a single physical cue responsible for the universal scale invariant phenomenon that the brain can exploit to optimize coding and perception of vocalized sounds.

## Introduction

Efficient coding strategies for representing natural sensory signals aim to generate compact neural representations of the external world. Barlow originally proposed the efficient coding hypothesis as a theoretical model of neural coding that aims to maximize information transfer between the external world and the brain while reducing metabolic and computational cost to an organism [1]. According to this model, neural computations performed by the brain should be optimized for extracting information from natural sensory signals and thus should be adapted for statistical regularities prevalent in natural environments.

One such statistical regularity is the widely-observed scale invariant relationship between the signal power and frequency of a sensory signal in which the power can be described as a power-law function of general form *S*_*xx*_(*f*) ∝ *f*^−*α*^, where *f* is the signal frequency and *α* is the scaling exponent. Natural visual scenes, for instance, exhibit this generalized form of scaling [2, 3] and it has been demonstrated that the spatial arrangement of object boundaries which contain edges are necessary to account for the empirically observed scaling exponent of *α* ≈ 2 [4–6]. Neurons in the central visual system are optimized to encode a wide range of edge orientations [3, 7], supporting the hypothesis that the brain is specialized for such statistical regularities in natural environments.

As for visual scenes, natural sounds also exhibit various forms of scale invariance, although the acoustic features that contribute to such phenomenon have remained elusive. Long-term fluctuations in the intensity profile of speech and music where first reported to exhibit scale invariance for frequencies < 1 Hz and with a scaling exponent of *α* ≈ 1 [8]. Subsequent studies further demonstrated that amplitude modulations of natural sounds including speech, animal vocalizations, environmental sounds also exhibit scale invariance [9–12]. Upon representing a natural sound by the analytic signal *S*_*A*_(*t*) = *x*(*t*)e^*iθ*(*t*)^, where $i=\sqrt{-1}$, *θ*(*t*) is the carrier phase, and *x*(*t*) is the modulation signal or equivalently the temporal envelope [13], the amplitude modulation power spectrum (AMPS) is defined as the Fourier transform magnitude of the envelope signal, *x*(*t*). For natural sounds the AMPS is well described by a generalized power-law function of the form *S*_*xx*_(*f*) ∝ *f*^−*α*^, such that the power in the envelope signal drops off with increasing modulation frequency (*f*) with exponent between *α* ≈ 1 − 2 within the approximate frequency range 1 to 100 Hz [10–12]. With the exception of water sounds, where scale invariance is accounted for by the distribution of self-similar acoustic “droplets” [9], it remains a mystery as to whether there are universal acoustic features that contribute to scale invariance for broader categories of natural and man-made sounds. Answering this question has important implications as neurons in the mammalian auditory system efficiently encode scale invariant structure in the sound envelope [12, 14] suggesting it is a critical driver of brain pathway function and perception abilities.

Physically, vocalization production in many species entails a source generator (e.g., vocal folds) that produces quasi-periodic envelope signal and articulatory gestures, for instance the opening and closing of the mouth and postural adjustments of the lips and tongue, that dynamically shape the sound envelope during speech production. Envelope fluctuations created by vocal fold vibration lie outside the modulation frequency range where scaling is observed [12] (i.e., >100 Hz) and thus should not contribute to scaling directly. In contrast, transient temporal onset and offset that mark the boundaries between isolated vocalizations are evident across many species and produce transient envelope fluctuations that may contribute to scaling behavior. In human speech, for instance, these salient features are generated by the time-dependent opening and closing of the oral cavity and related articulatory gestures. Drawing analogies from the statistics of natural visual scenes and the prevailing role of object boundaries [4, 5], we test the hypothesis that transient temporal edges account for the scaling phenomenon observed in natural vocalized sounds. We demonstrate that temporal edge boundaries in vocalizations are responsible for producing a amplitude modulation spectrum with dual-regime lowpass structure consisting of a flat region for low modulation frequencies and *f*^−2^ scale invariant trend at high modulation frequencies.

## Materials and methods

### Sound database

Sequences of vocalized sounds were obtained from a variety of digital sound sources. Vocalization sequences for a rat pup (HsdCpb/Wistar) [15], a mouse pup (C57BL/6 mice) [16], and a crying infant [17] all consisted of a single long-duration vocalization sequence (5–7 min duration; Table 1). Excerpts of continuously spoken speech totaling five minutes were obtained from a BBC broadcast reproduction of Hamlet [18]. Vocalization sequences were also obtained from various bird species [19] (Track 4: superb lyrebird, 35: winter wren, 41: common loon, and 46: gray-necked wood rail) and several species of new-world monkeys [20] (Track 9: Black Mantle Tamarin, Track 18: Golden Lion Tamarin, Track 32: White-Throated Capuchin Monkey, Track 43: Black Howler Monkeys, Track 48: Yellow Tail Wooly Monkey, Track 49: Common Wooly Monkey). Single long-duration sequences were not available for either bird or monkey vocalization categories and for this reason shorter sequences (monkey sequence range = 20–120 sec duration, average duration = 48 sec; bird sequence range = 26–135 sec, average duration = 60 sec) from different species were used to measure the envelope group statistics and AMPS for these groups. All of the vocalization sequence segments were selected because they contained well-isolated vocalization with minimal background noise. Vocalization sequences were sampled at a sampling rate (*F*_*s*_) to preserve the frequency content of each species (Table 1).

**Table 1:** Parameters used for envelope extraction and model fitting.

	Sound Duration (min)	*F*_*s*_ (kHz)	*DF*	*T*_*x*_	*f*_*low*_ (kHz)	*f*_*high*_ (kHz)
***Rat***	*6*	250	100	10	30	100
***Mouse***	*5*	300	100	30	30	100
***Bird***	*5*	44.1	10	30	0.5	20
***Monkey***	*9*.*66*	44.1	10	30	0.5	20
***Infant***	*7*	44.1	10	30	0.5	20
***Speech***	*5*	44.1	10	10	0.5	20

### Amplitude modulation power spectrum

For each vocalization sequence, we computed the vocalization sequence envelopes and computed the amplitude modulation power spectrum (AMPS) by extracting the temporal envelope of each sound sequence and subsequently computing the Fourier transform magnitude. Sounds were first bandpass filtered between frequencies *f*_*low*_ and *f*_*high*_ so as to encompass the frequency range of each vocalization sequence

$$s_{band}(t)=s(t)*h_{band}(t)$$

where *h*_*band*_(*t*) is a Kaiser bandpass filter impulse response (*β* = 5.6, filter order = 640, sidelobe error 60 dB) and * is the convolution operator. Since the vocalizations for each species has dominant energy over a unique frequency range, the frequencies *f*_*low*_ and *f*_*high*_ were individually selected based on visual inspection of the sound spectrum (Table 1). For the rat and mouse vocalizations the bandpass filter was selected to overlap the ultrasonic range (*f*_*low*_ = 30 kHz and *f*_*high*_ = 100 kHz) where the vocalizations had dominant energy. For the remaining vocalizations, *f*_*low*_ = 500 Hz and *f*_*high*_ = 20 kHz. *f*_*low*_ was chosen as 500 Hz because we measured the AMPS up to 250 Hz modulation frequency, which requires a carrier frequency of at least 500 Hz. The upper filter cutoff was selected as 20kHz which encompasses the bandwidth of the anti-aliasing filters for each recording.

For each of the bandpass filtered signals, we next extracted the envelope. This was done by first computing the analytic signal:

$$s_{A}(t)=s_{band}(t)+iH\{s_{band}(t)\}=x(t)e^{i\theta (t)}$$

where *H*{∙} is the Hilbert transform [13]. The temporal envelope is then obtained by taking the analytic signal magnitude

$$x(t)=|s_{A}(t)|.$$

**Fig 1:**
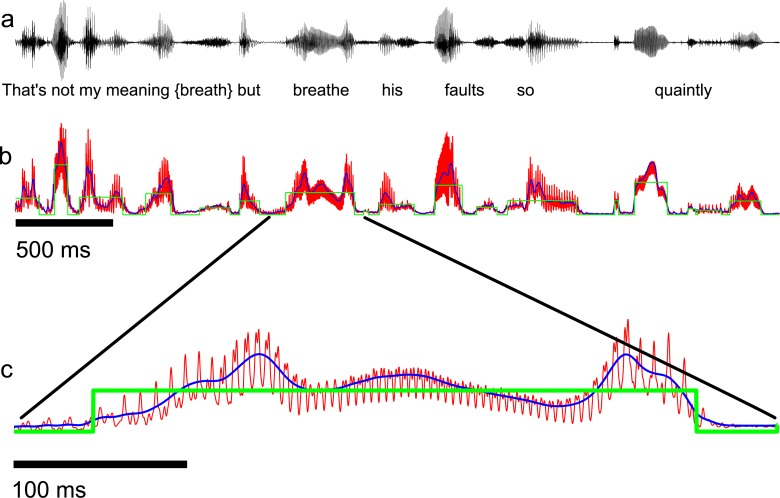
Envelope extraction, segmentation, and model fitting. (**a**) Acoustic waveform for a speech sample from the BBC reproduction of Hamlet containing the phrase “That’s not my meaning: but breathes his faults so quaintly.” (**b**) The envelope used for segmentation (blue) was obtained by lowpass filtering the analytic signal amplitude at 30 Hz whereas the envelope used for data analysis and model fitting was filtered at 250 Hz (red). The optimized model envelope for this example consists of sequence of non-overlapping rectangular pulses of variable duration and amplitude (green). (**c**) Zoomed-in view of a short segment of the corresponding envelopes in (**b**). The model (green) captures the transient onsets and offsets between consecutive speech elements and words, but is unable to capture other envelope features such as the fast-periodic fluctuations created through vocal fold vibration (~190 Hz fundamental in **c**) that are evident in the original envelope (red).

The envelopes were next passed through an antialiasing lowpass filter (250 Hz cutoff) to limit the modulation content to the range of interest (0–250 Hz), down sampled by a factor *DF* (see Table 1), and scaled for unit standard deviation. An example speech waveform excerpt and the corresponding filtered envelope obtained with the above procedure are shown in Fig 1 (Fig 1**A**, black = original sound waveform; Fig 1**B** and 1**C**, red = 250 Hz filtered envelope).

**Fig 2:**
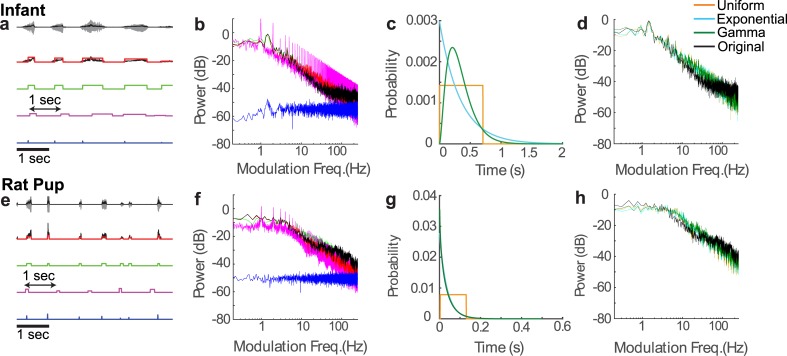
Relationship between sounds’ acoustic envelope parameters and AMPS illustrated for a crying infant and a rat pup vocalization sequences. (a and e) The original sound waveforms (gray line) and envelopes (black line) are shown along with the pulsed vocalization model (red line). Three models are also shown where one of the three parameters (amplitude, inter-vocalization interval, and duration) was perturbed. The perturbed pulse sequences have either constant pulse amplitudes (green), constant inter-vocalization intervals (magenta line), or zero durations (blue line). (b and f) Amplitude modulation power spectrum for original vocalization envelope and corresponding models (same color convention) show that manipulating durations has the most pronounced effect on the AMPS. (c and g) Vocalizations are also perturbed by synthetically modifying the duration distributions for infant (c) or rat (g) vocalization (uniform, exponential, or gamma distribution with matched mean and variance as the original vocalization). The duration distribution has minimal effect on the AMPS (d and h).

Finally, we computed the AMPS of each animal group. The power spectral density of the envelope, *x*(*t*), was estimated using a multi-taper spectral estimator (pmtm.m MATLAB function, NFFT = 16384, NW = 7/2). This procedure generates a power spectral density estimate with nominal frequency resolution of ~0.1 Hz. An NFFT value of 16384 was used to analyze all of the data with the exception the periodic simulated envelope of Fig 2 (magenta curves in Fig 2B and 2F). In order to achieve sufficiently high frequency resolution to resolve all of the envelope harmonics a value of NFFT = 262144 was used for this example.

### Vocalization sequence model

To test whether the envelope of isolated vocalizations contribute to the scale-invariant structure observed in vocalization sequences, we developed a stochastic vocalization sequence model consisting of a sequence of nonoverlapping rectangular pulses, *p*_*n*_(*t*). Each pulse marks the beginning and end of isolated vocalizations. The vocalization envelope can be approximated as

$$x(t)=\sum_{n=1}^{N}p_{n}(t)=\sum_{n=1}^{N}A_{n}∙rect\left(\frac{t-t_{n}}{D_{n}}\right)$$ (1)

where *n* is the pulse number and rect(∙) is a unit amplitude rectangular pulse with start time zero and 1 s duration. The number of isolated vocalizations within the *T* second interval is *N* ≅ *λT* where *λ* is the average vocalization rate in units of vocalizations/s. To account for the vocalization-to-vocalization variability in the sequence, pulse amplitudes (*A*_*n*_), onset times (*t*_*n*_) and durations (*D*_*n*_) are modeled as random variables. The envelopes from each vocalization sequence were fitted to the model of Eq 1 to assess how temporal sequence parameters (vocalization peak amplitudes, durations and onset times) contribute to 1/*f* structure. The fitting procedure consisted of two separate steps outlined in the following sections. This includes 1) a segmentation phase in which we detected and segmented the sequence into isolated vocalizations that stand out above the background noise level followed by 2) fitting the envelope from the segmented vocalization to rectangular pulses.

### Vocalization segmentation

In order to fit the vocalization sequence data for each animal group to the model of Eq 1, we first segmented the data into segments that contain single isolated vocalizations. Since isolated vocalizations occur at relatively low rates [21, 22] the envelopes of each vocalization sequence, *x*(*t*), were initially filtered to a maximum frequency *f*_*m*_ = 30 Hz with a 5-th order B-spline lowpass filter with continuously differentiable impulse response (differentiable to 5^th^ order) as shown for a speech segment (Fig 1**A**, black = original sound waveform; Fig 1**B**, blue = 30 Hz envelope). This 30 Hz lowpass filter is only applied during the vocalization segmentation phase and is used to identify sequence segments that contain isolated vocalizations (consisting of an onset and an offset component). Envelope segments that contained both an onset and offset were identified if the envelope exceeded a designated threshold level (*T*_*x*_, Table 1) above the envelope of sequence segments containing background noise. A short 2.7 sec long noise segment from each vocalization was identified audio-visually and used to measure the noise variance for each recording. The threshold level was set to 30 standard deviations (SD) above the noise floor for all vocalizations except for the rat and speech sequence (*T*_*x*_ = 10 SD) which required a lower threshold to minimize false negatives (i.e., vocalizations not detected by the algorithm as identified audio-visually). Using this approach total 2957 vocalization segments were identified (rat = 571, mouse = 492, bird = 518, monkey = 590, infant = 389, speech = 801).

### Vocalization model fitting

The model fitting procedure was performed on each isolated vocalization following the segmentation. During the fitting procedure, we used the signal envelopes that were lowpass filtered with a cutoff of 250 Hz (Fig 1**B**, red, shown for the speech segment in Fig 1**A**). Although it is theoretically possible to fit the vocalization model sequence of Eq 1 directly to the vocalization sequence envelope, the large number of parameters that would be required in the optimization are prohibitive. For instance, for the crying infant vocalization sequence there are *N* = 389 detected vocalizations, which would require that the algorithm optimize for a total of 1167 (389x3) model parameters. We tested such a global fitting procedure using least-squares and were unable to achieve convergence because of the high parameter dimensionality. Instead, we optimized for each of the detected vocalization sequence segments, which individually requires only three parameters. That is, for each detected vocalization segment, *x*_*n*_(*t*), we fitted a rectangular pulse, *p*_*n*_(*t*), of variable start time (*t*_*n*_), duration (*D*_*n*_), and peak amplitude (*A*_*n*_) using least-squares optimization. The optimization was carried out for all of the detected segments in each sequence. The results of this fitting procedure are illustrated for brief speech segment (Fig 1**B** and 1**C**, green). The model envelope accounts for the transient onsets and offsets that mark the beginning and end of vocalizations. It is not intended to model fast modulations that are also evident in the envelopes, such as those arising from periodic vocal fold vibration and which can be seen as a superimposed components (red) that ride on top of the slower vocalization envelope (blue) (zoomed in view in Fig 1**C**). The optimal parameters for each segment were then combined into three time-series (*t*_*n*_, *D*_*n*_ and *A*_*n*_) that were used to implement the full vocalization sequence model (Eq 1).

### Cutoff frequency estimation

For each of the vocalization AMPS, we empirically estimated the cutoff frequency, *f*_*c*_, where the AMPS transitions from a predominantly flat curve at low frequencies to a *f*^−2^ trend at higher frequencies. This was done by fitting the AMPS of each vocalization to a first-order lowpass spectrum model of the form

$$S_{xx}(f)=C/\left(f^{2}+f_{c}^{2}\right)$$

where *C* and *f*_*c*_ are free parameters to be determined. The estimated cutoff frequency was derived from the best fit solution of the first order model obtained numerically using least squares.

### Vocalization model AMPS derivation

In this section we derive a closed form solution for the AMPS of the stochastic vocalization sequence model of Eq 1. The modulation power spectrum of the vocalization model is obtained by taking the long-term expectation of the Fourier Transform Magnitude:

$$S_{xx}(f)=\underset{T\to \infty }{\lim}\frac{1}{T}E[X(f){X(f)}^{*}]$$

where *E*[∙] is the expectation operator taken across the three random variables (onset time, duration and amplitude),

$$X(f)=I\left\{x(t\right)\}=I\left\{\sum_{n=1}^{N}A_{n}∙rect(\frac{t-t_{n}}{D_{n}})\right\}=\sum_{n=1}^{N}A_{n}∙\frac{\sin\left(\pi D_{n}f\right)}{\pi f}e^{-j2\pi ft_{n}}$$

is the envelope Fourier transform (I{∙}), and * represents the complex conjugate. The model AMPS is then obtained as

$$\begin{array}{c} S_{xx}\left(f\right)=\underset{T\to \infty }{\lim}\frac{1}{T}E\left[X\left(f\right)X{\left(f\right)}^{*}\right]\\ =\underset{T\to \infty }{\lim}\frac{1}{T}E\left[\left(\sum_{n=1}^{N}A_{n}∙\frac{\sin\left(\pi D_{n}f\right)}{\pi f}e^{-j2\pi ft_{n}}\right)\left(\sum_{k=1}^{N}A_{k}∙\frac{\sin\left(\pi D_{k}f\right)}{\pi f}e^{+j2\pi ft_{k}}\right)\right]\\ =\underset{T\to \infty }{\lim}\frac{1}{T}E\left[\sum_{n=1}^{N}A_{n}^{2}∙\frac{\sin^{2}\left(\pi D_{n}f\right)}{\pi^{2}f^{2}}+\sum_{n=1}^{N}\sum_{k\neq n}A_{k}∙A_{n}∙\frac{\sin\left(\pi D_{n}f\right)}{\pi f}\frac{\sin\left(\pi D_{k}f\right)}{\pi f}e^{+j2\pi f(t_{k}-t_{n})}\right]. \end{array}$$

As will be illustrated subsequently the measured model parameters are largely independent and onset times are serially uncorrelated for the experimental data. This allows us to assume independence of the model parameters so that the second term inside the expectation approaches zero so that

$$S_{xx}(f)=\underset{T\to \infty }{\lim}\frac{1}{T}\sum_{n=1}^{N}E[A_{n}^{2}∙\frac{\sin^{2}\left(\pi D_{n}f\right)}{\pi^{2}f^{2}}]=\underset{T\to \infty }{\lim}\frac{N}{T}E\left[A_{n}^{2}∙\frac{\sin^{2}\left(\pi D_{n}f\right)}{\pi^{2}f^{2}}\right].$$

Since in the limiting case *λ* ≃ *N*/*T* and the random variables are approximately independent the AMPS simplifies as follows

$$S_{xx}(f)=\lambda ∙E[A_{n}^{2}]∙E\left[\frac{\sin^{2}\left(\pi D_{n}f\right)}{\pi^{2}f^{2}}\right]=\lambda ∙(\mu_{A}^{2}+\sigma_{A}^{2})∙E\left[\frac{\sin^{2}\left(\pi D_{n}f\right)}{\pi^{2}f^{2}}\right].$$

Finally, under the assumption that the vocalization durations are uniformly distributed within the interval [*T*_1_, *T*_2_]

$$\begin{array}{c} E\left[sin^{2}\left(\pi D_{n}f\right)\right]=\int p\left(\gamma \right)\;sin^{2}\left(\pi \gamma f\right)d\gamma \\ =\frac{1}{T_{2}-T_{1}}\int_{T_{2}}^{T_{1}}\sin^{2}\left(\pi \gamma f\right)d\gamma =\frac{1}{\left(T_{2}-T_{1}\right)∙2}\int_{T_{2}}^{T_{1}}1-\cos\left(2\pi \gamma f\right)d\gamma \\ =\frac{1}{2}∙\frac{1}{T_{2}-T_{1}}∙\left[T_{2}-T_{1}-\frac{\sin\left(2\pi T_{2}f\right)-\sin\left(2\pi T_{1}f\right)}{2\pi f}\right] \end{array}$$

so that the AMPS is

$$\begin{array}{c} S_{xx}(f)=\frac{\lambda ∙(\sigma_{A}^{2}+\mu_{A}^{2})}{{2∙\pi }^{2}f^{2}}∙\left[1-\frac{\sin(2\pi T_{2}f)-\sin\left(2\pi T_{1}f\right)}{(T_{2}-T_{1})∙2\pi f}\right]\\ =\frac{\lambda ∙(\sigma_{A}^{2}+\mu_{A}^{2})}{{2∙\pi }^{2}f^{2}}∙\left[1-\frac{T_{2}}{T_{2}-T_{1}}∙\mathrm{sinc}\left(2\pi T_{2}f\right)+\frac{T_{1}}{T_{2}-T_{1}}∙\mathrm{sinc}\left(2\pi T_{1}f\right)\right]. \end{array}$$

### Vocalization model cutoff frequency derivation

Given that the experimental and model AMPS both have lowpass structure, we derived in closed form the vocalization model AMPS cutoff frequency in order to relate this AMPS parameter to the vocalization model parameters (amplitude, duration and onset times). The vocalization model AMPS cutoff frequency (*f*_*c*_) is defined as the frequency where AMPS achieves half power (- 3dB) relative to the AMPS at zero frequency

$$S_{xx}(f_{c})={\frac{1}{2}∙S}_{xx}(0),$$

which for the model requires that the following equation be satisfied

$$\frac{\lambda ∙(\sigma_{A}^{2}+\mu_{A}^{2})}{{2∙\pi }^{2}f_{c}^{2}}∙\left[1-\frac{\sin(2\pi T_{2}f_{c})-\sin\left(2\pi T_{1}f_{c}\right)}{(T_{2}-T_{1})∙2\pi f_{c}}\right]=\frac{\lambda ∙(\sigma_{A}^{2}+\mu_{A}^{2})}{6(T_{2}-T_{1})}[T_{2}^{3}-T_{1}^{3}].$$

An approximate solution is obtained by noting that for large *f*_*c*_ > 1/2*π*(*T*_2_ − *T*_1_)

$$\frac{\sin(2\pi T_{2}f_{c})-\sin\left(2\pi T_{1}f_{c}\right)}{(T_{2}-T_{1})∙2\pi f_{c}}<\frac{1}{(T_{2}-T_{1})∙2\pi f_{c}}<1.$$

Considering this upper bound, the above equation is approximated as

$$\frac{1}{{2∙\pi }^{2}f_{c}^{2}}\approx \frac{1}{6(T_{2}-T_{1})}[T_{2}^{3}-T_{1}^{3}]$$

and solving for the cutoff frequency yields

$$f_{c}\approx \frac{1}{\pi }\sqrt{\frac{3∙(T_{2}-T_{1})}{\left[T_{2}^{3}-T_{1}^{3}\right]}.}$$

Finally, since *μ*_*D*_ = (*T*_1_ + *T*_2_)/2 and $\sigma_{D}^{2}={\left(T_{2}-T_{1}\right)}^{2}/12$ for a uniform distribution the cutoff can be expressed as

$$f_{c}\approx \frac{1}{\pi }\frac{1}{\sqrt{\mu_{D}^{2}+\sigma_{D}^{2}}}=\frac{1}{\pi }\frac{1}{\sqrt{E[D_{n}^{2}]}}.$$

## Results

### Temporal cues responsible for power-law scaling

We explore which temporal cues contribute to scaling phenomena in vocalization sequences. We consider a stochastic model of vocalization envelope sequence, *x*(*t*), containing three distinct forms of temporal variability (Eq 1, **Materials and methods**). The envelope of each vocalization sequence is approximated as a superposition of rectangular pulses each with a distinct onset time (*t*_*n*_), pulse amplitude (*A*_*n*_), and duration (*D*_*n*_). Each parameter is modeled as a random variable to account for vocalization-to-vocalization variability in the sequence.

Fig 2 illustrates how each of the model acoustic features contributes to the AMPS of natural vocalization sequences from an infant (a-d) and a rat pup (e-h), respectively. Vocalization amplitudes, onset times, and duration parameters are obtained for each vocalization in the sequence by fitting the model (a and e; red curve) to the original sound envelope (a and e; black curve) and the AMPS of the model envelope is computed (Fig 2B and 2F; see Materials and methods). Statistics for each of the estimated model parameters from the vocalization recordings is provided in Table 2 (see Materials and methods for details). The model AMPS (red) has a lowpass shape and power-law scaling similar to the original vocalization sequence AMPS (Fig 2B and 2F, black) with an RMS error of 3.9 dB (for frequencies between 1–100 Hz). Although the model follows the natural sound AMPS for low and intermediate modulation frequencies, it deviates at high modulation frequencies (Fig 2B, >100 Hz for infant; Fig 2F, >40 Hz for rat pup). In humans, this model disparity is partly explained by periodic modulations generated by the vocal fold vibrations [12] that contribute to the perceived vocal pitch and, though critical for identifying speech source attributes such as gender, they are not essential for speech intelligibility [23]. This result indicates that our model captures much of the general AMPS shape of natural vocalization sequences, particularly the power-law scaling trend.

**Table 2:** Estimated model parameters for each vocalization sequence.

* *	*μ*_*A*_	*σ*_*A*_	*μ*_*D*_ (s)	*σ*_*D*_ (s)	*μ*_*I*_ (s)	*σ*_*I*_ (s)	*λ*
***Rat***	0.86	2.14	0.036	0.039	0.63	0.82	1.59
***Mouse***	1.22	3.66	0.013	0.011	0.6	1.47	1.65
***Bird***	1.51	1.16	0.14	0.37	0.57	1.29	1.74
***Monkey***	1.14	0.86	0.14	0.21	0.53	0.76	1.89
***Infant***	1.58	1.02	0.33	0.2	1.06	0.99	0.94
***Speech***	0.84	0.72	0.22	0.27	0.37	0.36	2.68

Mean and standard deviation values are provided for the vocalization amplitude (*μ*_*A*_, *σ*_*A*_), duration (*μ*_*D*_, *σ*_*D*_) and inter-vocalization interval (*μ*_*I*_, *σ*_*I*_). *λ* is the vocalization rate (units of vocalizations/s).

By synthetically altering the model parameters we further explore how each temporal cue shapes the AMPS. First, we assess the contribution of vocalization amplitude variability by assigning a fixed amplitude to each model vocalization pulse (Fig 2A and 2E, green) while keeping all other parameters fixed. The pulse amplitudes are chosen so that the fixed amplitude model envelope and the original model envelope have matched variance. This manipulation has minimal effect on the AMPS (Fig 2B and 2F, green) since it maintains the lowpass shape and power-law scaling similar to the original vocalization sequence. Secondly, we manipulated the inter-vocalization intervals, defined as the time difference between consecutive vocalization onset times, Δ*t*_*n*_ = *t*_*n*+1_ − *t*_*n*_, to determine whether timing variability between vocalizations contributes to the power-law scaling. When we impose a constant inter-vocalization interval of 1 second (a and e, magenta) the modulation spectrum exhibits harmonic structure with 1Hz fundamental component that reflects the periodic structure of the inter-vocalization intervals. However, the resulting spectrum and the peak amplitude of the harmonics still follow the *f*^−2^ modulation spectrum trend (b and f, magenta), which suggest that the exact structure of the inter-vocalization intervals are not the critical parameters accounting for this behavior. Thirdly, temporal variation in vocalization durations is explored by replacing the pulse model approximation of each natural vocalization with a Dirac impulse that has a fixed duration of zero seconds (Fig 2A and 2E, blue). Removing the variation in vocalization duration results in a flat AMPS (Fig 2B and 2F, blue) that no longer exhibits scaling. The last manipulation conserves variations in the inter-vocalization intervals and amplitudes, indicating that these features alone are not sufficient to account for the lowpass trend with scaling at high frequencies whereas vocalization duration is critical.

To further explore the impact of vocalization durations we synthetically manipulated the duration distribution to determine how it contributes to scaling. We replaced the empirically measured durations with samples drawn from either a uniform (orange), exponential (light blue), or gamma (dark green) distribution with matched mean and variance (Fig 2C and 2G). As can be seen, the resulting AMPS is largely unaffected by the model distributions used as long as the vocalization durations have the same mean and variance (Fig 2D and 2H; as described subsequently). The measured RMS error between the simulated model AMPS with different duration distributions and the actual AMPS for modulation frequencies between 1–100 Hz was relatively small (between 3–4 dB for all of the distributions). This indicates that the AMPS shape is largely independent of the type of distribution used to model the vocalization durations.

**Fig 3:**
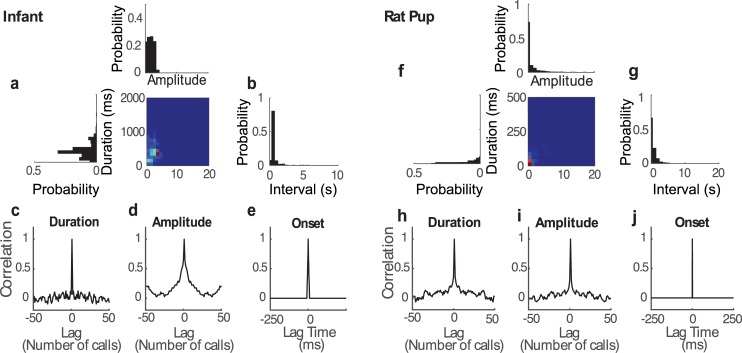
**Vocalization parameters and serial statistics for a crying infant (a-e) and rat pup call (f-j).** (a and f) Joint distribution of vocalization duration and amplitude is tightly distributed. The duration and amplitude marginal distributions are shown to the left and above the joint distribution. Inter-vocalization interval distributions (b and g) exhibit long exponential-like tails and a refractory region at short intervals. Serial statistics of the vocalization parameters exhibit weak temporal autocorrelation (c-e for a crying infant and h-j for rat pup call). Duration (c and h) and amplitude (d and i) parameters are largely serially uncorrelated. (e and j) Normalized autocorrelation for a point process consisting of onset times for each vocalization exhibits an impulsive autocorrelation.

It is conceivable that scaling emerges due to serial correlations and co-variation between the vocalization amplitudes, durations, and intervals. We assess these possibilities by examining the statistical structure of these three acoustic parameters for the infant and rat pup (Fig 3). The joint duration-amplitude distribution (Fig 3A and 3F) is relatively compact and these parameters exhibit a significant but weak correlation (infant, 0.11±0.04; rat, r = 0.49±0.05; mean±SE; t-test, p<0.01; see Table 3 for additional vocalization statistics). The autocorrelation for the duration and amplitude time series has impulsive structure, indicating minimal serial correlation for the infant and rat pup vocalization sequences (infant, Fig 3C and 3D; rat pup, Fig 3H and 3I). Furthermore, the inter-vocalization intervals follow an approximately exponential distribution as expected for a Poisson point process (Fig 3B and 3G), although there is a short latent period (~150 ms, infant; ~30 ms, rat pup) in the interval distribution indicating a brief silent period between consecutive vocalizations. Inter-vocalization intervals are weakly correlated with the vocalization duration and amplitude parameters (Table 3). Finally, upon treating the vocalization onset times as a renewal point process, we find that these are uncorrelated as evident from the impulse structure of the point process autocorrelation (Fig 3E and 3J). These analyses indicate that vocalization durations, amplitudes, and inter-vocalization intervals are distributed in a largely independent and serially uncorrelated fashion.

**Table 3:** Joint correlation statistics between the measured model parameters.

* *	*r*_*AD*_	*r*_*AI*_	*r*_*DI*_
***Rat***	0.49±0.05*	0.12±0.04*	0.18±0.04*
***Mouse***	0.21±0.06*	-0.05±0.01*	-0.05±0.04
***Bird***	0.10±0.02*	0.022±0.029	0.39±0.08*
***Monkey***	0.33±0.04*	0.08±0.03	0.1±0.05
***Infant***	0.11±0.04*	-0.20±0.03*	-0.01±0.05
***Speech***	0.37±0.03*	0.11±0.04*	0.08±0.03

Correlation statistics between the vocalization amplitudes (*A*), durations (*D*), and inter-vocalization intervals (*I*) are quantified using the Pearson correlation coefficient (mean±SEM). A significant correlation is noted by a * (bootstrap t-test, p<0.01).

### Amplitude modulation power spectrum of the stochastic model

To gain further insight on how each envelope parameter contributes to the scaling behavior in the AMPS, we derive the model AMPS in closed form by computing the power spectral density of the stochastic envelope model. Given that the estimated vocalization model parameters are weakly correlated (Fig 3 and Table 3), we assume independence of the model parameters to simplify the derivation. The model AMPS is (**Materials and methods,** Vocalization model AMPS derivation)

$$S_{xx}(f)=\lambda ∙E[A_{n}^{2}]∙E\left[\frac{\sin^{2}\left(\pi D_{n}f\right)}{\pi^{2}f^{2}}\right]=\lambda ∙(\mu_{A}^{2}+\sigma_{A}^{2})∙E\left[\frac{\sin^{2}\left(\pi D_{n}f\right)}{\pi^{2}f^{2}}\right]$$ (2)

where *E*[∙] is the expectation operator, $\mu_{A}^{2}$ and $\sigma_{A}^{2}$ are the amplitude mean-squared and variance, and $E[A_{n}^{2}]=\mu_{A}^{2}+\sigma_{A}^{2}$ is the second-order moment of *A*_*n*_. This result demonstrates that although the rate of vocalizations (*λ*) and amplitude statistics ($\mu_{A}^{2}+\sigma_{A}^{2}$) both affect the overall AMPS by a multiplicative gain factor, they do not depend on *f* and therefore do not affect the AMPS shape. Instead, the AMPS shape is primarily determined by the distribution of vocalization durations (term containing *E*[∙]). Since, as shown above, the exact duration distribution used has minimal impact on the AMPS shape (Fig 2D and 2H) we use a uniform distribution to simplify the analytic derivation. The AMPS is then evaluated in closed form as (**Materials and methods**, Vocalization model AMPS derivation)

$$S_{xx}(f)=\frac{\lambda (\mu_{A}^{2}+\sigma_{A}^{2})}{2\pi^{2}f^{2}}\left[1-\frac{T_{2}sinc(2\pi T_{2}f)-T_{1}sinc(2\pi T_{1}f)}{T_{2}-T_{1}}\right]$$ (3)

**Fig 4:**
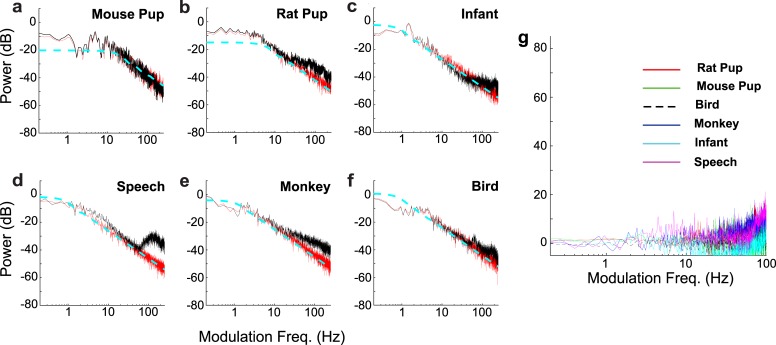
Comparison of AMPS from different species with the simulated model and the analytical solutions. AMPS (black) are shown for a mouse pup (a), rat pup (b), crying infant (c), speech (d), new world monkey (e), and bird (f) vocalizations. The simulated pulse vocalization model (red curves) has lowpass structure and 1/*f*^2^ trend at high frequencies that mirrors the scaling observed in the actual AMPS. The analytical solution likewise exhibits a lowpass structure with 1/*f*^2^ trend at high frequencies (Eq 3; dotted blue). (g) The residual error between the actual vocalization AMPS and simulated model AMPS lack the 1/*f*^2^ trend for different species.

Despite the simplifying assumptions, the analytic solution captures the general AMPS structure including the 1/*f*^2^ trend and the flat low frequency region for a human infant and rat pup vocalizations (Fig 4; actual AMPS, black; simulated AMPS, red; analytic solution AMPS, dotted blue).

Next, we evaluated the limiting AMPS behavior for these two regimes. For low frequencies (*f* → 0), it can be shown by applying L'Hospital's rule that:

$$S_{xx}(0)=\frac{\lambda (\mu_{A}^{2}+\sigma_{A}^{2})}{3}(T_{2}^{2}+T_{1}T_{2}+T_{1}^{2})$$ (4)

which is the limiting value in the flat low frequency AMPS region observed in Fig 4. By comparison, in the limiting case where the modulation frequency is large (i.e., *f* → ∞):

$$S_{xx}(f)=\frac{\lambda (\mu_{A}^{2}+\sigma_{A}^{2})}{2\pi^{2}}∙\frac{1}{f^{2}}$$ (5)

so that the AMPS behaves as a power-law for high *f* with a power-law exponent of *α* = 2. We find this dual regime lowpass structure is evident in all of the vocalization sequences examined (Fig 4). Although the model can deviate from the data as a result of vocalization production mechanisms not related to the temporal edges created by the initiation of isolated vocalizations (e.g., vocal fold vibration), in all cases the model captures the general lowpass structure. Furthermore, the model captures nearly all of the variability associated with the 1/*f*^2^ trend since the residual error spectrum lacks 1/*f*^2^ structure (Fig 4G) and all of the measured vocalizations sequence AMPS deviated from the simulated model by at most 3.9 dB (RMS error between model and data for frequencies between 1–100 Hz). This suggests that temporal edges are the main acoustic features accounting for the general scaling behavior.

Next, we explore the mechanism by which temporal edges in isolated vocalizations contribute to power-law scaling and the dual-regime structure. We start by noting that the vocalization sequence AMPS is precisely the average AMPS of individual vocalization envelopes if the vocalization onset times are serially uncorrelated. Considering the rectangular pulse vocalization sequence model (Eq 1), the AMPS of each rectangular pulse (*p*_*n*_(*t*)) is:

$$S_{p_{n}p_{n}}(f)=A_{n}^{2}∙D_{n}^{2}∙{sinc}^{2}(\pi D_{n}f)=A_{n}^{2}∙\frac{{sin}^{2}(\pi D_{n}f)}{\pi^{2}f^{2}}$$ (6)

**Fig 5:**
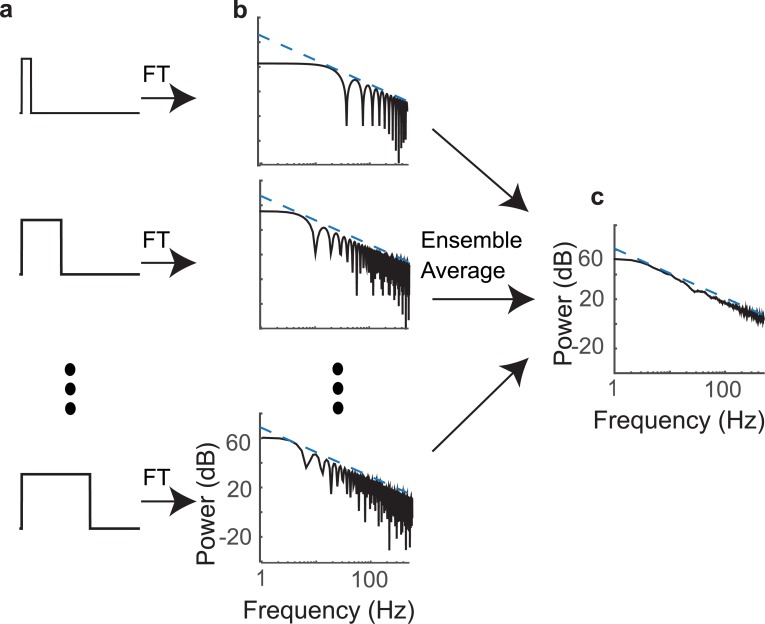
Ensemble averaging of vocalization pulse spectra predicts the observed vocalization AMPS. (a) Three example pulses from the speech ensemble. (b) The AMPS for each pulse consists of a sinc^2^ function with side lobe peaks and notch locations that depend on the vocalization duration and the side-lobe amplitudes that drop off proportional to 1/*f*^2^ (blue dotted lines). (c) The AMPS is obtained as the ensemble average across all durations, which produces an AMPS with lowpass structure and 1/*f*^2^ trend at high frequencies.

Thus, although isolated vocalizations contain both temporal onsets and offsets, which contribute to the 1/*f*^2^ behavior, on their own individual isolated vocalizations deviate from the 1/*f*^2^ trend. Based on Eq 6 the individual vocalization envelope power spectrum is approximated as a sinc^2^(∙) function with a spectrum amplitude proportional to the pulse amplitude squared and bandwidth that is inversely related to the pulse duration. This is evident from the power spectra (Fig 5B) of three exemplar rectangular pulses (Fig 5A) taken from the speech ensemble. The spectrum of a single pulse has a lowpass structure with oscillatory side-lobes that deviate from the 1/*f*^2^ trend (Fig 5B, black curves) although the peak amplitude of the side-lobes precisely follows the 1/*f*^2^ trend (blue curves).

We propose that the observed dual-regime 1/*f*^2^ structure arises from the collective averaging across an ensemble of isolated vocalizations in a sequence. As can be seen in Fig 3, isolated vocalizations have variable durations which consequently produce different notch and side-lobe configurations in the frequency domain (Fig 5B). Upon averaging the spectrum of each vocalization, notches and side-lobes interfere and cancel producing the 1/*f*^2^ regime. In contrast, the sinc^2^(∙) main lobes average constructively producing the flat AMPS regime at low frequencies. Thus, the dual-regime vocalization sequence AMPS behavior including the 1/*f*^2^ trend emerge naturally from the collective averaging across an ensemble of isolated vocalizations of variable durations.

### Tradeoff between vocalization duration and cutoff

As demonstrated in the simulations of Figs 2 and 5 and the closed form model derivations, the dual-regime lowpass structure of the vocalizations sequence AMPS likely arises through the superposition of spectra from isolated vocalizations each with a bandwidth that is inversely related to the vocalization duration. To determine the relationship between vocalization duration distribution and the transition point for the 1/*f*^2^ regime in the vocalization sequence AMPS, we derive the solution for the half power or cutoff frequency (*f*_*c*_) of the model AMPS (**Materials and methods**, Vocalization model cutoff frequency derivation). The analytic solution yields:

$$f_{c}\approx \frac{1}{\pi }∙\frac{1}{\sqrt{E[D_{n}^{2}]}}=\frac{1}{\pi }∙\frac{1}{\sqrt{\mu_{D}^{2}+\sigma_{D}^{2}}}$$ (7)

where *μ*_*D*_ and $\sigma_{D}^{2}$ are the duration mean and variance. This result indicates that the vocalization duration statistics are the primary determinants of the *f*_*c*_. Specifically, *f*_*c*_ is inversely related to the square root of the second order moment of the vocalization duration distribution. That is, vocalizations with a longer average duration will tend to have a lower *f*_*c*_ values while vocalizations with shorter durations will tend to have larger *f*_*c*_ values. This result is consistent with the results of (Fig 2A, 2B, 2E and 2F; blue curves) where we synthetically manipulated and set the vocalization model durations to zero. In such a case, vocalization pulses approach an impulse while the *f*_*c*_ approaches infinity and only the flat region of the AMPS is observed. This mathematical formulation is a statistical variant of the uncertainty principle for a vocalization ensemble, which requires that the signal duration in the time-domain be inversely related to its bandwidth in the frequency-domain [13].

**Fig 6:**
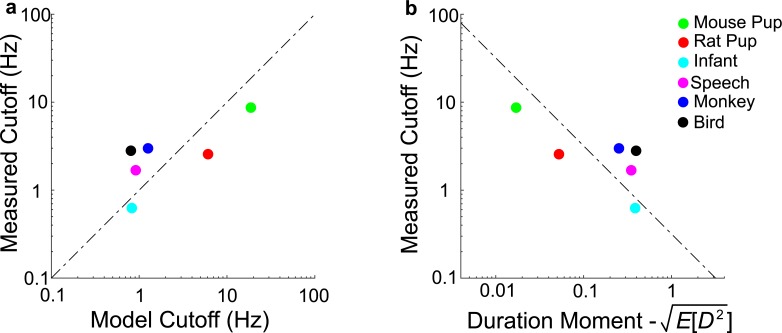
Time-frequency resolution tradeoff is predicted by the model. (a) The predicted cutoff frequencies from the vocalization duration statistics (Eq 7) for different vocalization recordings closely match the actual measurements. (b) Empirically measured *f*_*c*_ and duration second moment follow an inverse relationship as predicted by the model (Eq 7; dashed dot line).

Finally, we examine whether the measured durations from natural vocalization sequences can be used to predict *f*_*c*_ and therefore the transition point between the lowpass and 1/*f*^2^ regimes. As seen in Fig 6, the *f*_*c*_ estimated with our analytic model is correlated with the empirically measured *f*_*c*_ for the animal vocalization recordings examined (Fig 6A; log(*f*_*c*_) vs. log(*f*_*c*,*model*_), Pearson r = 0.76±0.24, mean±SEM; bootstrap t-test, p<0.05). Furthermore, measured *f*_*c*_ for the six recordings are inversely related to the experimentally measured second-order duration moment (Fig 6B; log(*f*_*c*_) vs. $log(E[D_{n}^{2}])$, Pearson r = -0.76±0.24, mean±SEM; bootstrap t-test, p<0.05) as predicted by Eq 7 (Fig 5, dotted line). This supports the idea that there is an inverse relationship between the vocalization durations and *f*_*c*_ that manifests as a tradeoff in time-frequency resolution.

## Discussion

The results describe for the first time a single physical cue that universally accounts for scale invariant phenomenon in the envelope of natural vocalization sequences from several animal recordings. We find that the ensemble of temporal boundaries or edges for isolated vocalizations is the principal determinant of power-law scaling relationship. In addition, we find a systematic inverse relationship between the average vocalization duration and frequency at which scaling behavior initiates (*f*_*c*_). Edges are responsible for the observed 1/*f*^2^ scaling region of the AMPS whereas the timing between consecutive on and off edges, which determine the vocalization duration, are critical in determining the flat region of the AMPS and the cutoff frequency (*f*_*c*_). These findings thus provide a new conceptual framework for characterizing the temporal statistics of natural vocalized sounds in terms of definable temporal cues within ongoing sound sequences. For example, one can conceptualize vocalization elements such as words and phonemes in speech as acoustic objects formed by temporal edges in the sound envelope and our study indicates that these are primary determinants of the temporal statistics captured in the AMPS of vocalization sequences. Moreover, temporal edges are perceptually salient [9, 24] and serve as temporal boundaries for grouping acoustic objects [25].

Although we have not extended our analysis to broader categories of sounds, other natural sounds [9–12] also exhibit scaling. The models and conceptual framework introduced here may have broad applicability as sound sequences and music in general are composed of transient and time-varying acoustic elements that can be coarsely modeled by onsets and offsets.

In vision, the spatial arrangement of object boundaries and the distribution of object size in opaque natural images all contribute to scale invariance [4–6]. In an analogous fashion, we have shown that vocalization boundaries consisting of edges in the time-domain likewise contribute to scaling in the acoustic realm. Importantly, isolated vocalizations are not sufficient since the 1/*f*^2^ trend arises from the collective averaging amongst an ensemble of vocalization with variable durations (Fig 5). Yet, unlike for natural scenes where the object size distribution needs to follow a power-law relationship, scaling for natural sounds does not depend critically on the exact vocalization duration distribution as long as the distributions have similar means (Fig 2 and closed form solutions). Furthermore, we point out that vocalization sequence onset times and amplitudes statistics are not critical to this result as determined from the closed form solutions of the model and demonstrated in Fig 2, where the model parameters where perturbed to constant values (periodic case for onset times and constant amplitude). Thus, the combined findings from the model and empirical perturbations provide strong evidence that the temporal edge boundaries in vocalizations are responsible for 1/*f*^2^ phenomenon.

In our analysis, we considered isolated vocalization sequences which have well-identified vocalizations and well-defined temporal boundaries. Whether similar results apply to more complex acoustic scenarios including natural soundscapes consisting of mixtures of vocalizations that are superimposed is unclear and needs to be determined. This is plausible given that images containing mixtures of translucent objects can also exhibit scale invariance [4, 5]. Previous works have demonstrated that although scaling is observed in natural environmental sounds, such sounds tend to have a scaling exponent that is somewhat lower than for vocalizations (scaling exponent closer to *α* = 1) [10, 11, 26]. One plausible hypothesis that needs to be considered is that background sounds often consist of mixtures of isolated sound, each of which has a well-marked onsets and offsets, so that the superposition of isolated acoustic objects could create phase distortions at the sound boundaries that distort temporal edges and ultimately have a whitening effect on the envelope AMPS, thus reducing the scaling exponent. Future studies need to explore how and if our findings can be generalized into a theoretical framework that applies to an even broader range of natural and man-made sounds.

Although the results provide a concise explanation for the 1/*f*^2^ scaling region that is linked to the temporal boundaries in vocalized sounds our model is not intended to account for other forms of scaling or features of the AMPS. Future studies and models are needed to further elucidate the acoustic generation mechanisms responsible for distinct regions of the AMPS of natural vocalized sounds. For instance, 1/*f* scaling has been previously described for very low modulation frequency (<0.1 Hz) for speech and music [8]. One plausible explanation for this phenomenon is that inter-vocalization statistics in sound sequences, such as for speech, have self-similar fractal structure at very long time scales [27] that may be responsible for 1/*f* scaling for very low modulation frequencies. Our model also is not intended to account for other features of the modulation spectrum, for instance the presence of periodic modulations created through vocal vibration and which are clearly evident in our speech envelopes and AMPS (Fig 1). These fast-periodic modulations are visible in the speech and infant vocalizations and show up as an additive modulation component in the AMPS (positive AMPS deflection above the expected model results). A recent study observed the presence of peaks in the modulation spectrum of speech and music in the vicinity of 3–5 Hz [28] and lacked 1/*f*^2^ structure described here. This difference is due to the fact that the calculation of the modulation spectrum in that study used modulation filters with logarithmic bandwidths that mimic neural modulation tuning functions [12] to estimate the modulation power. Applying such modulation filters magnifies the output power by a factor proportional to *f*, so that the flat region of the AMPS we describe increases proportional to *f* and the region containing the 1/*f*^2^ trend decreases proportional to 1/*f*. Consequently, a peak is observed in the modulation spectrum within the vicinity of the cutoff frequency (*f*_*c*_) where the transition between the flat and 1/*f*^2^ behavior is observed in our model. We have confirmed the observations of Ding et al. by estimating modulation spectrum with octave band filters or alternately multiplying the modulation spectrum by *f* as described (S1 Fig). In both cases, the resulting modulation spectrum contain a primary peak in the vicinity of ~3 Hz as observed by Ding et al., but we also observe a secondary peak within the vicinity of 100–300 Hz where vocal fold vibration is prominent. Ding et al did not observed such a peak because they characterized the modulation power spectrum only up to 32 Hz.

The results have a number of implications for theories of coding by the brain since auditory neurons are exquisitely sensitive to temporal transitions with millisecond precision [29–31] and have been shown to produce an efficient neural representation that equalize the modulation power of natural sounds [12, 14]. Similar strategies have been proposed in vision where neurons through edge detection equalize or “whiten” the spectrum of natural images enabling an equitable use of neural resources [3]. Mechanistically, two distinct temporal coding mechanisms could contribute to such efficient representation in audition. First, auditory neurons have excitatory-inhibitory (on-off) responses to temporal edges that effectively perform a smooth temporal derivative operation on the sound envelope [32–35]. In the time domain, this could facilitate temporal edge detection for important information bearing acoustic temporal elements, analogous to edge detection in vision [3, 7]. In the frequency domain, such temporal derivative operation has a transfer function squared-magnitude *H*^2^(*f*) = 4*π*^2^*f*^2^ that opposes and precisely cancels the 1/*f*^2^ scaling of natural sounds thus whitening the spectrum. Secondly, power equalization could be partly achieved through modulation filter bandwidth scaling as previously observed for auditory midbrain neurons [12] and perceptually [36]. For both neurons and perception, modulation filter bandwidths increase proportional to *f*. This bandwidth scaling magnifies the output power by *f*, partly canceling the 1/*f*^2^ power trend observed for natural sounds [12] (as shown in S1 Fig). In combination, temporal edge detection and bandwidth scaling could provide mechanisms to equalize modulation power in vocalizations allowing for efficient information transfer and coding, analogous to principles in vision.

The findings are also relevant for sound coding and hearing technologies. For instance, the stochastic framework could be used to improve coding, compression, and sound recognition algorithms. The findings could further be used to improve algorithms to enhance detection of transient sound elements [37] in order to facilitate recognition in hearing aid, cochlear implant, and other assistive hearing technologies.

## Supporting information

S1 Fig Speech AMPS obtained using a proportional resolution modulation filter bank that mirrors neural modulation filters in the auditory midbrain (Rodriguez et al 2010). The AMPS of speech derived with constant quality factor modulation filters (Q = 1, black curve) exhibits a primary peak within the rhythm perceptual range at ~3 Hz. A secondary peak is observed at ~150 Hz which corresponds to the temporal modulations created through vocal fold vibration. The green curve corresponds to the speech AMPS after multiplying by the modulation frequency (*f)*.
(EPS)
